# σ_2_R/TMEM97 in retinal ganglion cell degeneration

**DOI:** 10.1038/s41598-022-24537-3

**Published:** 2022-12-01

**Authors:** Hua Wang, Zhiyou Peng, Yiwen Li, James J. Sahn, Timothy R. Hodges, Tsung-Han Chou, Qiong Liu, Xuezhi Zhou, Shuliang Jiao, Vittorio Porciatti, Daniel J. Liebl, Stephen F. Martin, Rong Wen

**Affiliations:** 1 Bascom Palmer Eye Institute, University of Miami, Miller School of Medicine, Miami, FL 33136 USA; 2 Department of Chemistry and Biochemistry, University of Texas at Austin, Austin, TX 78712 USA; 3 Department of Biomedical Engineering, Florida International University, Miami, FL 33174 USA; 4 Department of Neurosurgery, University of Miami, Miller School of Medicine, Miami, FL 33136 USA

**Keywords:** Neuroscience, Cell death in the nervous system

## Abstract

The sigma 2 receptor (σ_2_R) was recently identified as an endoplasmic reticulum (ER) membrane protein known as transmembrane protein 97 (TMEM97). Studies have shown that σ_2_R/TMEM97 binding compounds are neuroprotective, suggesting a role of σ_2_R/TMEM97 in neurodegenerative processes. To understand the function of σ_2_R/TMEM97 in neurodegeneration pathways, we characterized ischemia-induced retinal ganglion cell (RGC) degeneration in *TMEM97*^*−/−*^ mice and found that RGCs in *TMEM97*^*−/−*^ mice are resistant to degeneration. In addition, intravitreal injection of a selective σ_2_R/TMEM97 ligand DKR-1677 significantly protects RGCs from ischemia-induced degeneration in wildtype mice. Our results provide conclusive evidence that σ_2_R/TMEM97 plays a role to facilitate RGC death following ischemic injury and that inhibiting the function of σ_2_R/TMEM97 is neuroprotective. This work is a breakthrough toward elucidating the biology and function of σ_2_R/TMEM97 in RGCs and likely in other σ_2_R/TMEM97 expressing neurons. Moreover, these findings support future studies to develop new neuroprotective approaches for RGC degenerative diseases by inhibiting σ_2_R/TMEM97.

## Introduction

Sigma receptors were originally identified by radioactive ligand-binding assays as a subclass of opioid receptors^1^, but subsequent studies indicated that they were a distinct group of receptors with two members: the sigma-1 receptor (σ_1_R) and the sigma-2 receptor (σ_2_R)^2–5^. σ_1_R was cloned in 1996, and the protein sequence showed no homology to any other mammalian protein^6^. On the other hand, the molecular nature of σ_2_R remained elusive until 2017 when a membrane protein previously known as transmembrane protein 97 (TMEM97) was purified using a resin coupled with a high-affinity σ_2_R-binding ligand^7^. The ligand-binding properties of TMEM97 match those of the enigmatic σ_2_R, so σ_2_R was finally identified as TMEM97^7^. The structure of σ_2_R/TMEM97 and its complexes with several ligands have been recently reported^8^, and 20(*S*)-hydroxycholesterol (20(*S*)-OHC) has been identified as an endogenous σ_2_R/TMEM97 ligand^9^.

The biological function of σ_2_R/TMEM97 is not well understood, but its wide distribution in the body, including the brain, the liver, pancreas, testis, and other organs^10–12^, implies that it may have distinct functions in different cells. σ_2_R/TMEM97 is known to regulate intracellular Ca^2+^ levels^13^ and to play a role in cholesterol trafficking and uptake^14–16^. The high expression of σ_2_R/TMEM97 in proliferating cancer cells makes it a biomarker and target for cancer diagnosis and therapy^17–23^. σ_2_R/TMEM97 is also involved in various neurological disorders^24^. Recent studies in animal models of Alzheimer's disease and traumatic brain injury demonstrate that small molecules binding selectively to σ_2_R/TMEM97 are neuroprotective^25,26^, suggesting that σ_2_R/TMEM97 is involved in pathways relevant to neurodegeneration.

Toward understanding the role of σ_2_R/TMEM97 in neurodegenerative processes, we characterized ischemia-induced retinal ganglion cell (RGC) degeneration in mice with or without σ_2_R/TMEM97 (i.e.,* TMEM97*^+*/*+^or *TMEM97*^*−/−*^ mice). Herein we report that in *TMEM97*^*−/−*^ mice, the absence of σ_2_R/TMEM97 renders RGCs resistant to ischemia-induced cell death. Moreover, intravitreal injection of a selective, high-affinity σ_2_R/TMEM97 ligand DKR-1677 protects RGCs from ischemia damage in wildtype (*TMEM97*^+*/*+^) mice. These findings demonstrate that σ_2_R/TMEM97 facilitates RGC degeneration. Our present work is a breakthrough in understanding the biological function of σ_2_R/TMEM97 in neurodegenerative processes in RGCs and likely in other *TMEM97* expressing neurons. Importantly, these results support the development of novel therapeutic approaches to prevent or slow the progression of RGC degeneration by inhibiting the function of σ_2_R/TMEM97.

## Results

### Retinas of *TMEM97*^*−/−*^ mice

**Figure 1:**
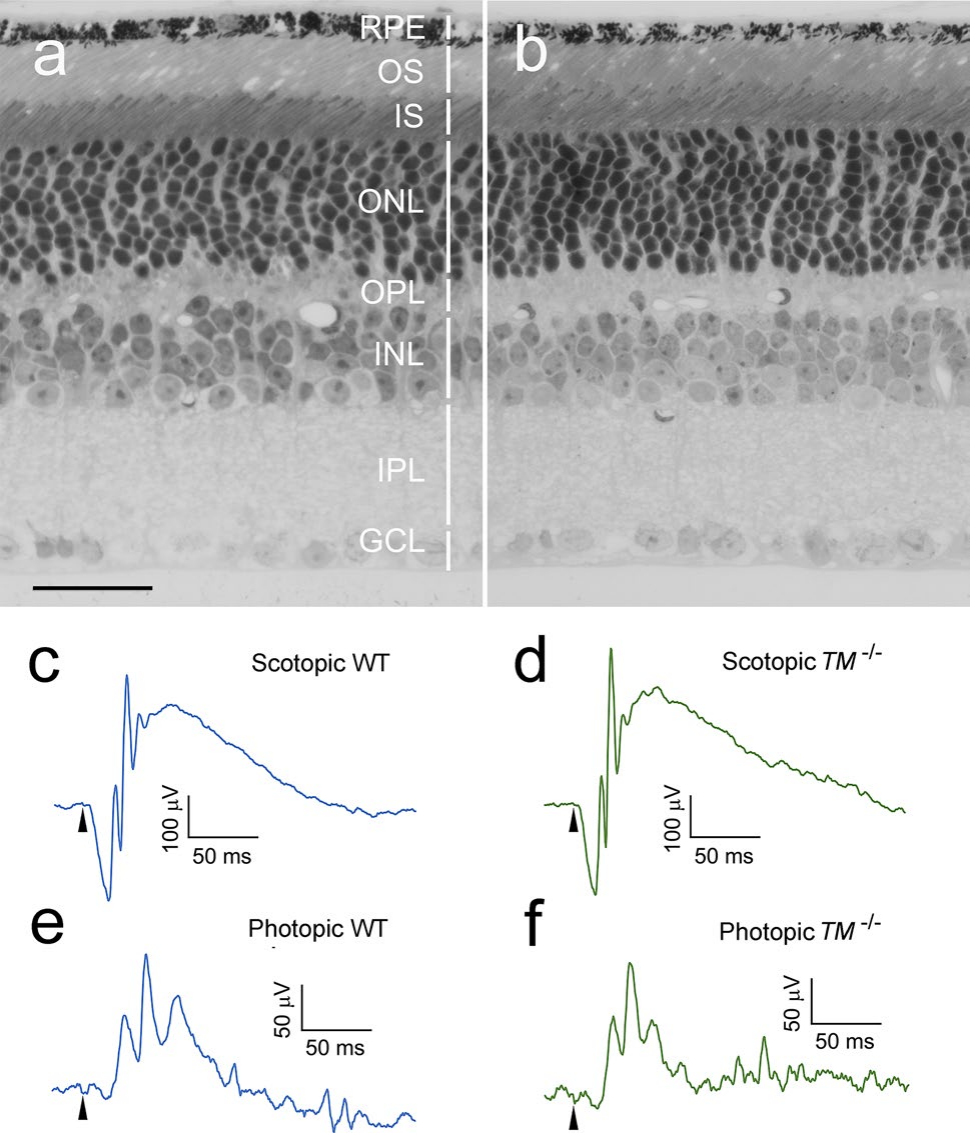
Retinal morphology and ERGs of *TMEM97*^*−/−*^ mice. A semi-thin retinal section of a *TMEM97*^*−/−*^ mouse (**b**) shows well-organized retinal layers indistinguishable from the structure of the wildtype mouse (**a**) (retinal layers are indicated by white vertical bars). Scotopic ERGs (evoked by flashes of 0.60 log cd s/m^2^) and photopic b-waves (elicited by flashes of 1.20 log cd s/m^2^) from the *TMEM97*^*−/−*^ (*TM*^*−/−*^) mice (**d**,**f**, respectively) are very similar to scotopic and photopic ERGs from wildtype (WT) mice (**c**,**e**, respectively). *RPE* retinal pigment epithelium, *OS* photoreceptor outer segments, *IS* photoreceptor inner segments, *ONL* outer nuclear layer, *OPL* outer plexiform layer, *INL* inner nuclear layer, *IPL* inner plexiform layer, *GCL* ganglion cell layer. Scale bar: (**a**,**b**) 30 µm. The onset of the flash is indicated by arrowheads in (**c**–**f**).

*TMEM97*^*−/−*^ mice are viable and fertile with no visible gross abnormalities. The retinal structure of a *TMEM97*^*−/−*^ mouse (Fig. 1b) is indistinguishable from the retina of a wildtype (WT) animal (Fig. 1a). Scotopic and photopic ERGs (electroretinograms) from the *TMEM97*^*−/−*^ mice (*TM*^*−/−*^, Fig. 1c,d, respectively) are similar to those from the wildtype mice (WT, Fig. 1e,f, respectively). In both *TMEM97*^*−/−*^ mice and wildtype mice, scotopic b-waves were reliably recorded with flash intensity as low as − 4.4 log cd s/m^2^. The visual function of the *TMEM97*^*−/−*^ mice was further assessed by measuring the visual acuity (VA) in an optomotor system^27^. The average VA of *TMEM97*^*−/−*^ mice is 0.45 ± 0.024 cpd (cycle per degree, n = 4, Fig. S1), which is within the reported range of wildtype mice^28^.

**Figure 2:**
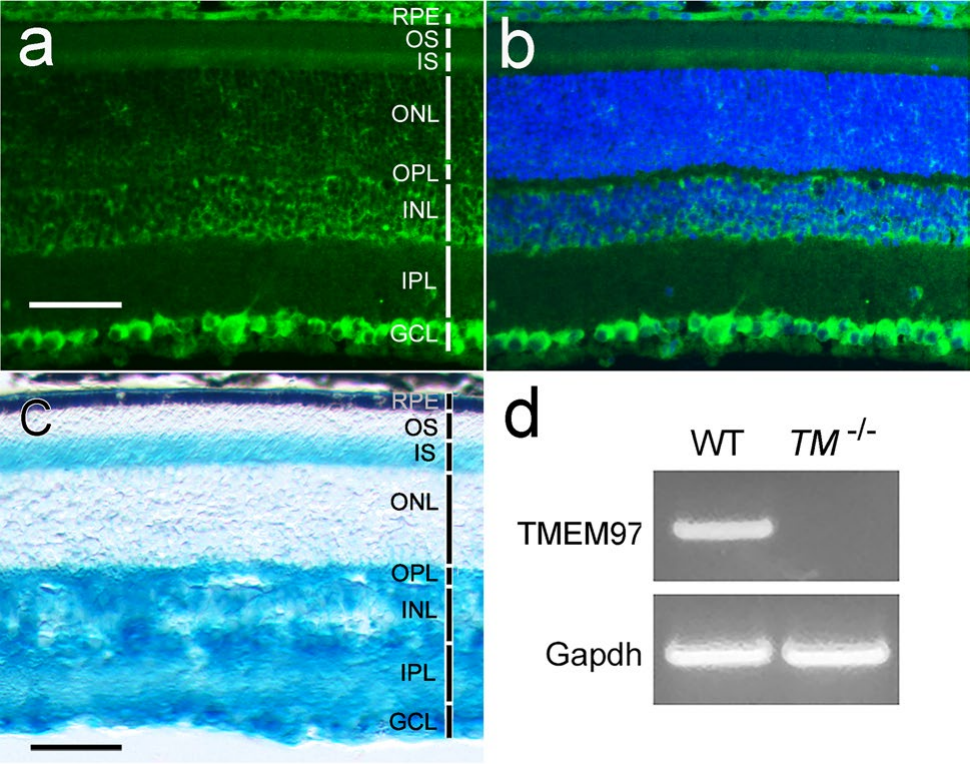
σ_2_R/TMEM97 expression in RGCs in the retina. σ_2_R/TMEM97 immunoreactivity in the retina of a wildtype mouse was detected in RGCs as well as in the RPE; the IS, sparsely in the ONL; cells in the INL (**a**). The same retinal section in (**a**) is shown in (**b**) with DAPI staining for cell nuclei. In the retina of a *TMEM97*^*−/−*^ mouse (**c**), X-gal blue staining is present in the RGCs, and in the IS, the ONL (sparsely), cells in INL, the IPL, and the OPL. The blue stain in the RPE is mostly masked by melanin pigment (**c**). Retinal layers are indicated by white (**a**) or black (**c**) vertical bars. The RNA expression of *TMEM97* in the retina was examined by RT-PCR (**d**). Robust *TMEM97* expression (TMEM97) was detected in the retinas of the wildtype animals (**d**, WT) but absent in the retinas of *TMEM97*^*−/−*^ mice (**d**, *TM*^*−/−*^). Expression of Gapdh served as a reference (**d**). Scale bars (**a**–**c**): 50 µm. The full gel image in (**d**) is displayed in Fig. S3.

In the retina of a wildtype mouse, σ_2_R/TMEM97 immunoreactivity was found in RGCs, the photoreceptor inner segments (IS), the RPE (retinal pigment epithelium), cells in the inner nuclear layer (INL), and sparsely in the outer nuclear layer (ONL) (Fig. 2a,b). No σ_2_R/TMEM97 immunoreactivity was detected in the retina of the *TMEM97*^*−/−*^ mouse (Fig. S2).

The expression of *TMEM97* in RGCs was also confirmed by X-gal staining in the *TMEM97*^*−/−*^ mice. The *TMEM97* gene in *TMEM97*^*−/−*^ mice was replaced with a Velocigene cassette ZEN-Ub1 that has a *lacZ* reporter gene under the control of the *TMEM97* promoter^29^, so the expression of the *lacZ* reporter correlates with *TMEM97* expression. Blue X-gal staining is present in the RGCs as well as in the IS, the INL, the outer plexiform layer (OPL), the inner plexiform layer (IPL), the RPE, and sparsely in the ONL (Fig. 2c). In the RPE cells, the blue staining is mostly masked by the heavy melanin pigment in the 20 µm thick section (Fig. 2c).

The σ_2_R/TMEM97 distribution pattern in the wildtype mouse (Fig. 2a) and the X-gal staining pattern in the *TMEM97*^*−/−*^ mouse overlap with each other, except in the IPL and OPL where σ_2_R/TMEM97 immunoreactivity is negative but blue X-gal stain is present. This discrepancy could be caused by differences in the subcellular localization of σ_2_R/TMEM97 and β-galactosidase because σ_2_R/TMEM97 is an ER membrane protein, but β-galactosidase is not.

The absence of *TMEM9*7 expression in the retinas of *TMEM97*^*−/−*^mice was confirmed by reverse transcription PCR (RT-PCR). Robust *TMEM97* mRNA expression was found in the wildtype retinas (WT, Fig. 2d, Fig. S3), but no *TMEM97* mRNA was detected in the retinas of *TMEM97*^*−/−*^ mice (*TM*^*−/−*^, Fig. 2d, Fig. S3)*.*

### Ischemia-induced RGC degeneration in *TMEM97*^*−/−*^ mice

**Figure 3:**
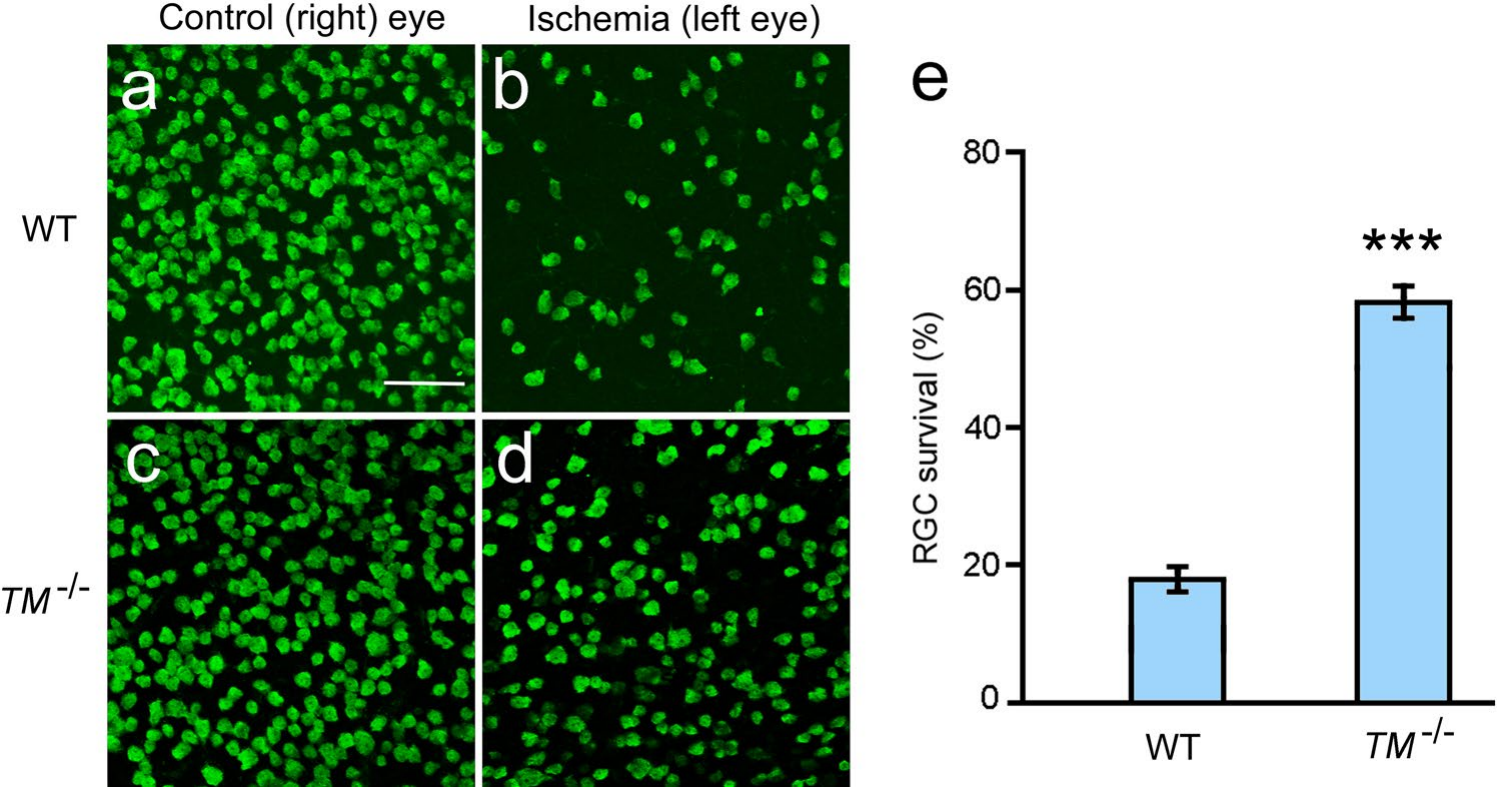
RGC survival in *TMEM97*^*−/−*^ mice after retinal ischemia. RGCs were identified as RBPMS-positive cells in the flat-mounted retinas (**a**–**d**). A significant loss of RGCs is apparent in the left eye of a wildtype (WT) mouse (**b**) after ischemia as compared with the untouched fellow (right) eye of the same animal (**a**). In the retina of a *TMEM97*^*−/−*^ mouse (*TM*^*−/−*^), many RGCs are present after ischemia (**d**). The retina of the untouched fellow (right) control eyes from the same animal in (**d**) is shown in (**c**) for comparison. RGC survival rates were calculated and presented in (**e**). The survival rate of *TMEM97*^*−/−*^ mice is significantly higher than that of the wildtype control mice (**e**). Scale bar: 100 µm. Triple asterisks indicate P < 0.001.

Retinal ischemia was induced by elevating the intraocular pressure (IOP) to 120 mm Hg for 45 min in the left eyes of both wildtype and *TMEM97*^*−/−*^ mice. The right eyes were untouched and served as naïve controls. Eyes were collected seven days after ischemia, and RGCs were identified by immunostaining of the RGC marker RBPMS (RNA binding protein with multiple splicing)^30^. A significant loss of RGCs was observed in the left eyes of wildtype mice as compared to RGCs in the untouched right eye (Fig. 3a,b). In contrast, many RGCs are present in the left eyes of *TMEM97*^*−/−*^ mice after retinal ischemia (Fig. 3d). For comparison, the retina of the naïve fellow eye from the same animal in Fig. 3d is shown in Fig. 3c. The RGC survival rate was calculated as the ratio of RBPMS-positive cells in the left eye *vs* those in the right eye in the same animal. In the *TMEM97*^*−/−*^ mice, the average RGC survival rate is 0.59 ± 0.02 (n = 9), which is significantly higher than the average rate of 0.18 ± 0.01 (n = 8, P < 0.001) in the wildtype animals (Fig. 3e). These results demonstrate that RGCs in adult animals lacking σ_2_R/TMEM97 are resistant to ischemia-induced cell death.

The mean RGC density in the naïve eyes of the *TMEM97*^*−/−*^ mice (271 ± 7/0.1 mm^2^, n = 9) is significantly lower than the average RGC density in the wildtype mice (366 ± 15/0.1 mm^2^, n = 8, P < 0.001). In contrast, the mean RGC density in eyes of *TMEM97*^*−/−*^ mice after ischemia (157 ± 7/0.1 mm^2^, n = 9) is much higher than that in the eyes of wildtype animals after ischemia (65 ± 4/0.1 mm^2^, n = 8, P < 0.001). The lower RGC density in the naïve eyes of the *TMEM97*^*−/−*^ mice is consistent with the lower baseline mean amplitude of PERG (pattern electroretinogram) from the naïve eyes in the *TMEM97*^*−/−*^ mice (see below).

### Pattern ERG measurement

PERG has been used as a sensitive measurement to assess the electrophysiological function of RGCs^31^. The PERG amplitude was measured peak-to-trough from the peak of the major positive wave at about 80 ms to the trough of the major negative component at about 300 ms. PERG latency was the time-to-peak of the major positive wave at about 80 ms. To monitor changes in RGC electrophysiological activities, we first recorded baseline PERG (Pre-IS) in wildtype and *TMEM97*^*−/−*^ mice. The left eyes in each of these groups of animals were then subjected to ischemia, and the PERG was measured again seven days after ischemia (Post-IS).

**Figure 4:**
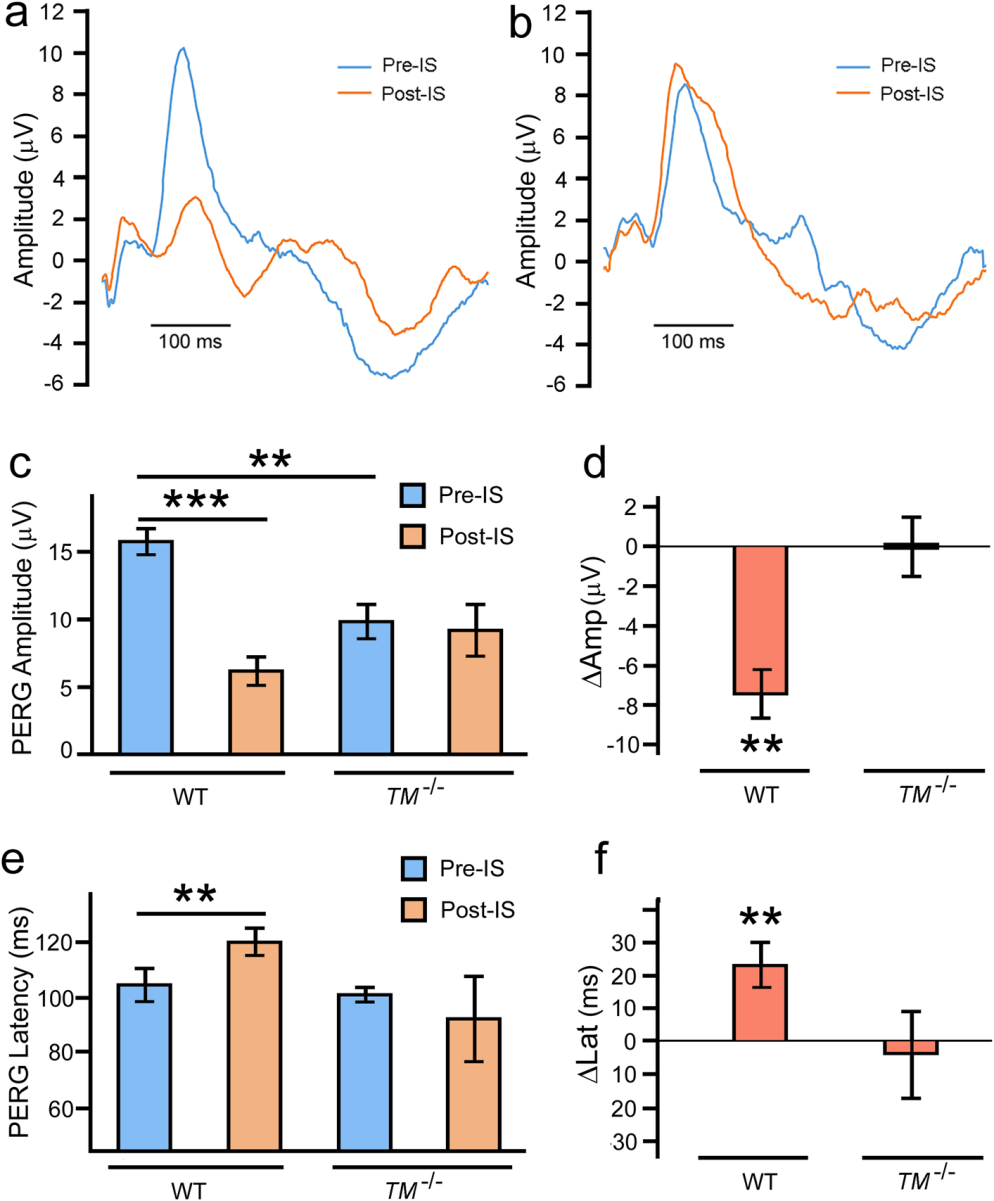
PERG measurements. Representative PERG recordings from a wildtype mouse show a significant decrease in PERG amplitude and an increase in PERG latency after retinal ischemia (**a**, Post-IS) as compared to the baseline amplitude of the same eye (**a**, Pre-IS). No significant change in PERG amplitude or latency was found in PERG recordings from the *TMEM97*^*−/−*^ mouse (**b**, Pre-IS and Post-IS). The mean PERG amplitude showed a dramatic decrease in the wildtype mice after ischemia (**c**, WT, Post-IS) as compared to the baseline (**c**, WT, Pre-IS). No significant change in mean PERG amplitude was seen in *TMEM97*^*−/−*^ mice (**c**, *TM*^*−/−*^, Pre-IS and Post-IS). The change in PERG amplitude in each animal (∆Amp, Post-IS minus Pre-IS) was calculated and the average ∆Amp is significantly different from zero in WT mice (**d**, WT) but not in *TMEM97*^*−/−*^ mice (**d**, *TM*^*−/−*^). There was also an increase in mean PERG latency in wildtype mice after ischemia (**e**, WT), but not in *TMEM97*^*−/−*^ mice (**e**, *TM*^*−/−*^). The change in latency in each animal (∆Lat, Post-IS minus Per-IS) was calculated and the average ∆Lat is significantly different from zero in wildtype mice (**f**, WT), but not in *TMEM97*^*−/−*^ mice (**f**, *TM*^*−/−*^). The baseline mean PERG amplitude in the *TMEM97*^*−/−*^ mice (**c**, *TM*^*−/−*^, Pre-IS) is lower than that in the wildtype mice (**c**, WT, Pre-IS). Double asterisks indicate P < 0.01. Triple asterisks indicate P < 0.001.

In the retina of the wildtype mouse, ischemia markedly reduced PERG amplitude (Fig. 4a**,** Post-IS) as compared to the baseline (Fig. 4a**,** Pre-IS). No such change was observed in PERG amplitude following ischemia in the retina of the *TMEM97*^*−/−*^ mouse (Fig. 4b**,** Pre-IS and Post-IS). A significant ischemia-induced decrease in mean PERG amplitude is seen in wildtype animals (Fig. 4c, WT ~ 60%, P < 0.001, n = 5), but not in the *TMEM97*^*−/−*^ mice (Fig. 4c, *TM*^*−/−*^, n = 5). Ischemia-induced change in PERG amplitude in each animal was calculated as ∆Amp (Post-IS minus Pre-IS) to show that the average ∆Amp is significantly different from zero in the wildtype mice (P < 0.01, n = 5) (Fig. 4d**,** WT, n = 5). In contrast, the average ∆Amp in *TMEM97*^*−/−*^ mice is not significantly different from zero (Fig. 4d, *TM*^*−/−*^, n = 5).

Retinal ischemia also induced an increase in PERG latency in the retina of the wildtype mouse (Fig. 4a), but not in the retina of the *TMEM97*^*−/−*^ mouse (Fig. 4b). A significant increase in mean PERG latency (~ 20 ms, P < 0.01, n = 5) (Fig. 4e, WT, Post-IS) is observed in the wildtype mice as compared with the baseline (Fig. 4e WT, Per-IS), but there is no significant increase in mean PERG latency in *TMEM97*^*−/−*^ mice (Fig. 4e, *TM*^*−/−*^, Per-IS and Post-IS). Ischemia-induced change in PERG latency in each animal was calculated as ∆Lat (Post-IS minus Per-IS) to show that the average ∆Lat in wildtype mice is significantly different from zero (P < 0.01, n = 5) (Fig. 4f), whereas the average ∆Lat in *TMEM97*^*−/−*^ mice is not significantly different from zero (Fig. 4f, *TM*^*−/−*^, n = 5).

The baseline mean PERG amplitude in the *TMEM97*^*−/−*^ mice (Fig. 4c, *TM*^*−/−*^, Pre-IS) is significantly lower (~ 30%, P < 0.01, n = 5) than that in wildtype mice (Fig. 4c, WT, Pre-IS), which is consistent with the lower RGC density in the *TMEM97*^*−/−*^ mice.

### Protection of RGCs by DKR-1677

**Figure 5:**
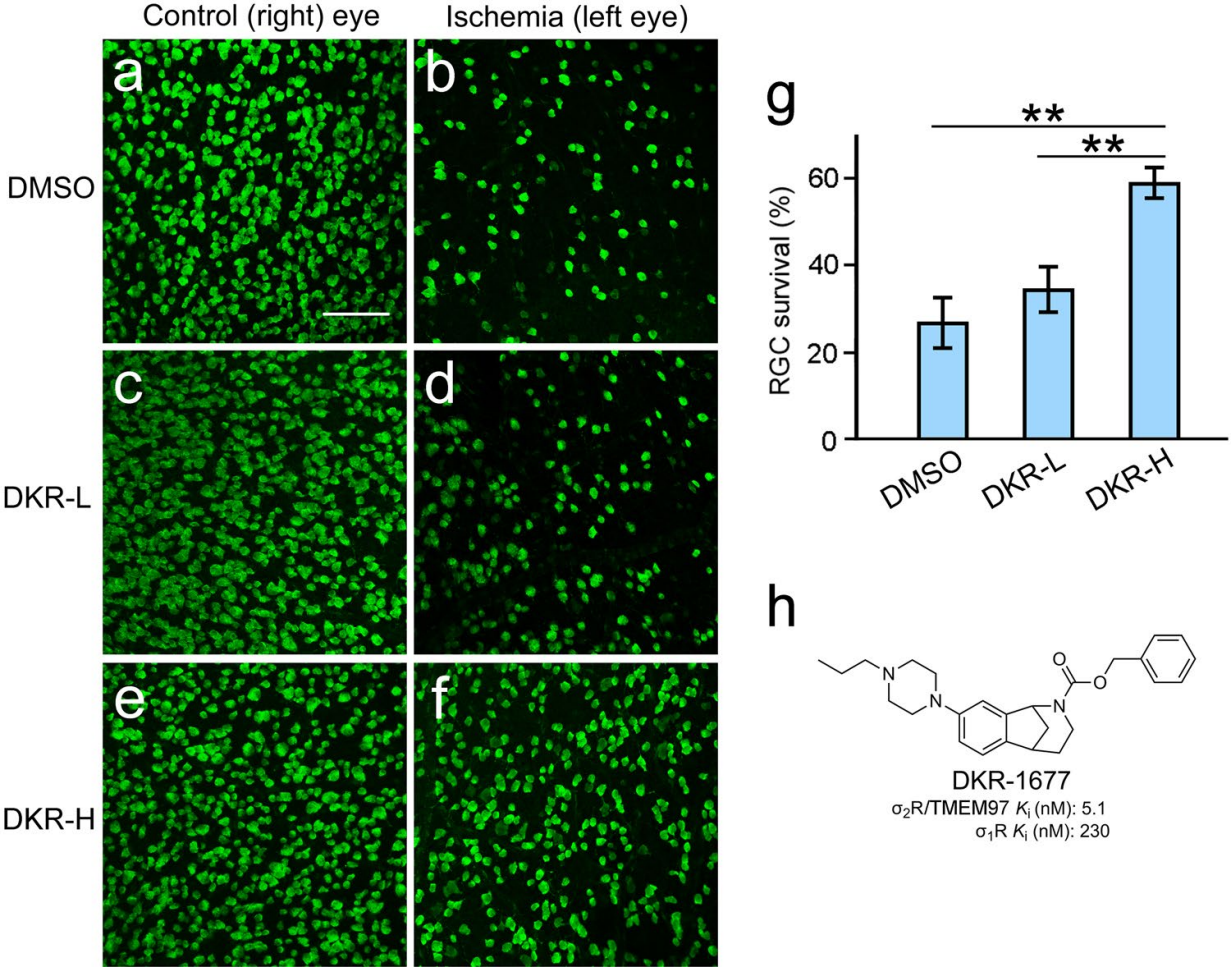
RGC protection by DKR-1677. Eyes of wildtype mice were intravitreally injected with DKR-1677 or DMSO and then subjected to ischemia. There was a significant loss of RGCs in the DMSO treated left eye after ischemia (**b**) as compared with the untouched fellow (right) eye of the same animal (**a**). Similar RGC loss was found in the eyes treated with a low-dose of DKR-1677 (DRK-L) (**d**), as compared to the right control eye of the same animal (**c**). In the eye treated with a high-dose of DKR-1677 (DRK-H),  many RGCs remained after ischemia (**f**). The retina of the untouched fellow (right) eye of the same animal in (**f**) is shown in (**e**) for comparison. The RGC survival rate in the DKR-H group (**g**) is significantly higher than that in the DKR-L and DMSO group, respectively (**g**). The structure of DKR-1677 and its binding affinity for σ_1_R (guinea pig) and σ_2_R/TMEM97 (rat) are presented in (**h**). Scale bar (**a**–**f**): 100 µm. Double asterisks (**g**) indicate P < 0.01.

The neuroprotective effect of a σ_2_R/TMEM97 ligand DKR-1677 on RGCs was examined in wildtype mice. DKR-1677 is a selective and high-affinity σ_2_R/TMEM97 ligand (Fig. 5h) that has been shown to be neuroprotective in two models of traumatic brain injury^26^. For intravitreal injections, DKR-1677 was dissolved in DMSO (dimethylsulfoxide) at concentrations of 2 µg/µL and 20 µg/µL. The left eyes were injected with 2 µL of either the low-dose (2 µg/µL) or the high-dose (20 µg/µL) formulation of DKR-1677. The vehicle control animals were injected with 2 µL DMSO into the left eyes. The right eyes of animals in the DKR-1677 and DMSO groups were not injected. Retinal ischemia was performed on the left (injected) eyes five days after injection, and the right eyes were untouched. RGC survival was assessed seven days after ischemia.

A significant loss of RGCs was seen in the left eyes in the vehicle group after ischemia, as compared with the untouched right eyes (Fig. 5a,b). Similar cell loss was found in the retina treated with low-dose DKR-1677 (DKR-L) as compared with the control right eye (Fig. 5c,d). In eyes injected with high-dose DKR-1677 (DKR-H), many surviving RGCs were present (Fig. 5e). For comparison, the retina of the naïve fellow eye from the same animal in Fig. 5f is shown in Fig. 5e. The average RGC survival rate in eyes treated with high-dose DKR-1677 (DKR-H, 0.59 ± 0.08, n = 5) is significantly higher than that in vehicle controls (DMSO, 0.27 ± 0.02, n = 5, P < 0.01) and eyes treated with low-dose DKR-1677 (DKR-L, 0.34 ± 0.03, n = 4, P < 0.01), respectively (Fig. 5g). The difference in RGC survival rate between the DKR-L and DMSO groups is not statistically significant (Fig. 5g).

The mean RGC densities in the naïve eyes of three groups (DMSO, 377 ± 15, n = 5; DKR-L, 360 ± 13, n = 4; DKR-H, 351 ± 55/0.1 mm^2^ n = 5) are not significantly different. On the other hand, the mean RGC density in eyes treated with high-dose of DKR-1677 and received ischemia (209 ± 18/0.1 mm^2^, n = 5) is significantly higher than that in the eyes subjected to ischemia in DMSO control animals (102 ± 18/0.1 mm^2^, n = 5, P < 0.01), or in the eyes treated with low-dose of DKR-1677 and received ischemia (123 ± 20/0.1 mm^2^, n = 4, P < 0.05). The difference in RGC densities between the DKR-L and DMSO groups is not statistically significant. The retinal overall structure showed no visible changes after injection of high-dose DKR-1677 (Fig. S4). These results demonstrate that the σ_2_R/TMEM97 ligand DKR-1677 protects RGCs against ischemia-induced cell death in a dose-dependent manner.

## Discussion

A significant discovery of the present work is the loss-of-function phenotype in RGCs in the *TMEM97*^*−/−*^ mice. When challenged by retinal ischemia, the RGCs in *TMEM97*^*−/−*^ mice had significantly higher survival rates than the RGCs having fully functional σ_2_R/TMEM97 in wildtype mice. Moreover, electrophysiological activities of RGCs were much better maintained after ischemia in *TMEM97*^*−/−*^ mice than in wildtype mice. These findings unequivocally demonstrate a role of σ_2_R/TMEM97 in the degenerative processes to facilitate ischemia-induced RGC degeneration. Another important finding is the neuroprotective effect of DKR-1677 against ischemia related RGC death in wildtype mice. The effect of DKR-1677 resembles the loss-of-function phenotype in RGCs in *TMEM97*^*−/−*^ mice, indicating that the binding of DKR-1677 to σ_2_R/TMEM97 inhibits its function. Thus, the mechanism of action of DKR-1677, and perhaps other neuroprotective σ_2_R/TMEM97 ligands, is inhibiting the function of σ_2_R/TMEM97.

Selective σ_2_R/TMEM97-binding compounds have been reported to be neuroprotective in other studies. For example, DKR-1677, the σ_2_R/TMEM97 ligand used in the present work, has been shown to be neuroprotective in two models of traumatic brain injury^26^. Moreover, SAS-0132, a compound structurally similar to DKR-1677, was found to significantly improve cognitive performance in a mouse model of Alzheimer's disease^25^. Furthermore, a σ_2_R/TMEM97-binding compound CT1812 is in clinical trials for treating Alzheimer's disease^32^.

Intriguingly, the mean RGC density in the *TMEM97*^*−/−*^ mice is lower than that in the wildtype mice. It is known that in early development of the retina, excessive RGCs are generated, and many undergo programmed cell death before the final number of RGCs is reached^33,34^. The lower RGC density in the *TMEM97*^*−/−*^ mice is evidence that σ_2_R/TMEM97 is perhaps involved in the mechanism to determine the final RGC numbers during retinal development. This finding has important implications, and future studies will elucidate the role of σ_2_R/TMEM97 in regulating RGC numbers during early development.

RGCs express both σ_1_R and σ_2_R/TMEM97. σ_1_R has been shown to promote RGC survival^35^, and genetic ablation of *σ*_*1*_*R* in mice leads to an age-related inner retinal dysfunction and loss of RGCs^36^. σ_1_R agonists have also been shown to protect RGC^35,37^. We show herein that σ_2_R/TMEM97 facilitates RGC degeneration. It seems that the two receptors play opposite roles in RGC degeneration. The question remains as to whether σ_1_R and σ_2_R/TMEM97 interact with each other to regulate RGC degeneration.

The biological functions of σ_2_R/TMEM97 are not well understood, although σ_2_R/TMEM97 is known to be involved in regulating cellular Ca^2+^ levels^13^ and controlling cellular cholesterol levels^14–16^. It has long been recognized that treating cells with σ_2_R/TMEM97 ligands induces a biphasic increase in cytosolic Ca^2+^ levels^13^, indicating that σ_2_R/TMEM97 signaling could be mediated by intracellular Ca^2+^ levels. σ_2_R/TMEM97 is also known to regulate cellular cholesterol by controlling cholesterol trafficking from lysosomes to the ER and cholesterol uptake^14–16^. For example, a trimeric complex of σ_2_R/TMEM97, the progesterone membrane component 1 (PGRMC1), and the low-density lipoprotein (LDL) receptor has been shown to regulate cholesterol levels in cells by internalizing LDL^16^. A recent study identified 20(*S*)-OHC as an endogenous σ_2_R/TMEM97 ligand^9^. 20(*S*)-OHC binding to σ_2_R/TMEM97 significantly enhances the interaction between σ_2_R/TMEM97 and NPC1 (NPC intracellular cholesterol transporter 1), providing evidence that 20(*S*)-OHC regulate cholesterol trafficking from lysosomes to the ER^9^.

σ_2_R/TMEM97 is shown to be associated with several neurological disorders, including anxiety, depression, and addiction^24^. In animal models, σ_2_R/TMEM97 binding compounds are known to mitigate alcohol withdrawal symptoms^38,39^ and the effects of cocaine^40^, as well as to induce long-lasting relief of neuropathic pain^41^. A potential role of σ_2_R/TMEM97 in oxidative damage to RPE cells has been reported in two papers, but the results are contradictory^42,43^. Further studies could clarify the potential role of σ_2_R/TMEM97 in oxidative damage in RPE cells.

Our finding that inhibiting σ_2_R/TMEM97 protects RGCs could have specific clinical relevance. RGCs are output neurons that process and convey visual information from the retina to the visual cortex^44^. Several clinical conditions, including glaucoma, ischemic optic neuropathies, hereditary optic neuropathies, and demyelinating disease, lead to RGC death, optic nerve damage, and blindness^45–47^. Glaucoma is a heterogeneous group of optic neuropathies characterized by RGC degeneration and a leading cause of irreversible vision loss^48–51^. A promising therapeutic strategy for treating glaucoma is to protect RGCs^46,52^. The present work provides strong experimental data supporting a new neuroprotective approach for RGCs by inhibiting σ_2_R/TMEM97. Small molecules like synthetic σ_2_R/TMEM97 ligands have the advantage over biological agents of being able to penetrate the blood-retinal barrier. Accordingly, they are excellent candidates for the development of neuroprotective agents for treating glaucoma and other degenerative retinal diseases.

In summary, the present work is a breakthrough toward elucidating the function of σ_2_R/TMEM97 in RGCs and other σ_2_R/TMEM97 expressing neurons. We show that σ_2_R/TMEM97 facilitates RGC degeneration and that inhibiting σ_2_R/TMEM97 function is neuroprotective. This work also provides persuasive experimental support for a new therapeutic strategy to protect RGCs and other neurons in neurodegenerative diseases by inhibiting σ_2_R/TMEM97.

## Methods

### Ethics statement

All procedures involving animals complied with the ARRIVE (Animal Research: Reporting of In Vivo Experiments) guidelines and adhered to the ARVO (Association for Research in Vision and Ophthalmology) Statement for the Use of Animals in Ophthalmic and Vision Research, and were approved by the Institutional Animal Care and Use Committee of University of Miami, Miller School of Medicine.

### Animals

Wildtype mice (C57Bl/6) were purchased from Jackson Labs (Bar Harbor, ME). *TMEM97*^*−/−*^ mice (*Tmem97*^*tm1(KOMP)Vlcg*^, stock # 050147-UCD) were obtained from the Mutant Mouse Resource & Research Centers (MMRRC, https://www.mmrrc.org/), backcrossed and maintained on a C57BL/6 background. Genotypes were confirmed by PCR according to MMRRC instructions. Animals were kept on a 12:12 light–dark cycle. Adult male mice (2–3 months old) were used in all experiments.

### Histology

For retinal histology, animals were killed by CO_2_ overdose and immediately followed by vascular perfusion with mixed aldehydes^53,54^. Eyes were collected and embedded in an Epon/Araldite mixture. Semi-thin sections (1 µm) were cut to display the entire retina along the vertical meridian^53,54^. Retinal sections were stained with toluidine blue and examined by light microscopy.

### ERG recording

ERGs were recorded with an UTAS system (LKC Technologies, Gaithersburg, MD). Animals were anesthetized with intraperitoneal ketamine/xylazine (80/10 mg/kg). Pupils were dilated with 0.1% atropine and 0.1% phenylephrine HCl. During recording, animals were maintained at 37 °C on a heating pad. A contact lens electrode was placed on the cornea, a reference electrode under the skin of the forehead, and a ground wire electrode was placed under the skin close to the base of the tail. ERGs were elicited by 1 ms white flashes in the Ganzfeld sphere. Animals were dark adapted for > 3 h before scotopic ERG recording. Photopic ERG responses were recorded with a low white background illumination (30 cd s/m^2^). Inter-stimulus intervals were 10 s. Each recording was an average of 5 responses.

### Optomotor response measurement

Visual acuities were assessed with an optomotor system (OptoMotryAT, Cerebral Mechanics, Lethbride, Alberta, Canada) by observing optomotor responses of the animal to rotating sinusoidal gratings^27^. An animal was placed on the platform and tested by a staircase protocol with an initial spatial frequency of 0.1 cpd and 0.05 cpd step size at 100% contrast and a drift speed of 12.0 degree/sec. The test protocol was controlled by OptoMotryAT software (version 3.0.4.). The highest spatial frequency that a mouse could respond to was taken as the visual threshold or visual acuity of that mouse.

### Immunocytochemistry

For TMEM97 immunostaining, animals were killed by CO_2_ overdose. Eyes were removed after vascular perfusion with 4% paraformaldehyde, cryoprotected with 20% sucrose, and embedded in Tissue-Tek OCT compound (Miles Inc., Elkhart, IN). Sections (10 µm) along the vertical meridian were cut on a Cryostat at − 20 °C and thaw-mounted onto Super Frost Plus glass slides (Fisher Scientific, Pittsburgh, PA). Retinal sections were incubated with anti-TMEM97 antibodies (PA5-23003, Thermo Fisher Scientific Technology, Waltham, MA) at 4 °C overnight. TMEM97 immunoreactivity was visualized by staining with Cy2 conjugated secondary antibodies (Jackson ImmunoResearch Labs, West Grove, PA) for 1 h at room temperature (RT). Cell nuclei were stained with DAPI (4',6-diamidino-2-phenylindole). Fluorescent signals in the retinal sections were examined by confocal microscopy (LSM700; Carl Zeiss, Jena, Germany).

### X-gal stain in the retina

*TMEM97*^*−/−*^ mice were generated by replacing 8169 base pairs of the *TMEM97* gene in chromosome 11 (positions 78,355,984–78,364,152) with a Velocigene cassette ZEN-Ub1 that has a *lacZ* reporter gene under the control of the *TMEM97* promoter (https://www.mmrrc.org/catalog/sds.php?mmrrc_id=50147)^29^. The expression of *lacZ* (encoding β-galactosidase) thus correlates with the expression of *TMEM97* and the enzymatic activity of β-galactosidase can be detected by X-gal staining. For X-gal staining in the retinas of *TMEM97*^*−/−*^ mice, eyes were collected and fixed in 2% paraformaldehyde and 0.2% glutaraldehyde. Retinal cryo-sections (20 µm) were cut and stained with a phosphate-buffered saline (PBS) containing 0.5 mg/mL X-gal (5-bromo-4-chloro-3-indolyl β-D-galactopyranoside), 5 mM potassium ferricyanide, 5 mM potassium ferrocyanide, 2 mM MgCl_2_, 0.01% sodium deoxycholate, and 0.02% Nonidet P-40 (NP-40) at 37 °C overnight. Stained retinal sections were examined by DIC (differential interference contrast) microscopy.

### TMEM97 RNA expression

*TMEM97* mRNA expression in wildtype and *TMEM97*^*−/−*^ mice was examined by reverse transcription PCR (RT-PCR). Total RNA was extracted from four retinas of *TMEM97*^*−/−*^ or wildtype mice with the RNeasy kit (Qiagen, Germantown, MD). cDNA was synthesized using 2 µg total RNA as the template and poly d(T) (deoxythymidine) primer with the ProtoScript II cDNA synthesis kit in a 20 µL reaction (New England Biolabs, Ipswich, MA). TMEM97 expression was examined by PCR with forward primer 5’-TCTACTTCGTCTCGCACATCCC-3’ and reverse primer 5’-CCGGCAGCTTCCTTTGAAGAAGG-3’. The expression of the housekeeping gene *Gapdh* (Glyceraldehyde-3-phosphate dehydrogenase, forward primer 5’-AGGTTGTCTCCTGCGACTTC-3’ and reverse primer 5’-GGGTGGTCCAGGGTTTCTTAC-3’) was used as a reference. PCR was carried out using 2 µL cDNA reaction as the template with OneTaq DNA polymerase (New England Biolabs). The total volume of PCR reaction (30 µL) was electrophoresed on a 2% agarose gel and PCR products were visualized by ethidium bromide staining.

### Retinal ischemia

Retinal ischemia was induced by elevating the intraocular pressure (IOP) to 120 mm Hg. An animal was anesthetized with isoflurane through a nose cone and body temperature was kept at 37 °C with a heating pad. The anterior chamber of the left eye was cannulated with a 33-gauge needle connected to a reservoir of saline (0.9% NaCl) via a valve. The reservoir was placed at 163 cm above the eye to create a pressure of 163 cm H_2_O (equivalent to 120 mm Hg). IOP was elevated to 120 mm Hg (163 cm H_2_O) by turning the valve to connect the needle to the reservoir. Ischemia was visually confirmed as the eye turned pale. Elevated IOP was maintained for 45 min, whereupon perfusion to the eye was resumed by turning the valve off to disconnect the needle from the reservoir followed by needle withdrawal.

### Quantification of RGC survival

RGCs were identified by immunostaining of RBPMS^30^. Eyes were collected seven days after ischemia. The anterior segment of each eye was removed, and the eyecup was incubated with anti-RBPMS antibodies (GTX118619, GeneTex, Irvine, CA) at 4 °C overnight. The eyecup was then washed and stained with Cy2 conjugated secondary antibodies (Jackson ImmunoResearch Labs) for 1 h at RT to visualize RBPMS immunoreactivity. After staining, the retina was cut into four quadrants (superior, inferior, nasal, and temporal), flat-mounted onto glass slides, and examined by confocal microscopy (LSM700; Carl Zeiss).

RBPMS positive cells in each quadrant were counted in five fields (319 × 319 µm each) at 0.5 mm (1 field), 1 mm (2 fields), and 1.5 mm (2 fields) from the optic nerve head, and RGC densities were calculated. RGC survival rate is the ratio of RBPMS positive cells in the left eye *vs* those in the right eye.

### Pattern electroretinogram (PERG) recording

PERG was recorded as previously described^55,56^. Mice were anesthetized with ketamine/xylazine (80/10 mg/kg) and gently restrained in an animal holder. Animals were kept at a constant body temperature of 37 °C using a feedback-controlled heating pad (TCAT-2LV; Physitemp Instruments, Inc. Clifton, NJ). Pupils were undilated and small (< 1 mm) to ensure a large depth of focus. PERG was recorded with a JÖRVEC system (JÖRVEC, Miami, FL) using a stainless-steel subcutaneous needle electrode (Grass, West Warwick, RI) placed in the snout. The reference and ground electrodes were identical needles placed in the medial portion of the scalp and at the root of the tail, respectively. Visual stimuli were contrast-reversing bars (0.05 cycles/deg, 98% contrast, 800 cd/m^2^ mean luminance) generated on a light-emitting diode tablet (15 × 15 cm size) and presented to the eye at a 10 cm distance. PERG responses were analyzed by automated detection of the main positive peak (P1) at about 80 ms, and the subsequent negative trough (N2) at about 300 ms. PERG amplitude was defined as the amplitude difference between the P1 peak and the N2 trough. PERG latency was defined as the time-to-peak of the major positive wave at about 80 ms.

### Intravitreal injections

The selective σ_2_R/TMEM97 ligand DKR-1677 was prepared as previously described^57^ and dissolved in DMSO at two concentrations of 2 µg/µL and 20 µg/µL. A 33-gauge needle connected to a 10-µL microsyringe (Hamilton, Reno, NV) was used for intravitreal injections as described^58^. The left eye of a mouse was injected with 2 µL of DKR-1677 (2 µg/µL or 20 µg/µL) and the right eyes received no injection. In the vehicle controls, the left eyes were injected with 2 µL DMSO, and the right eyes were not injected.

### Statistics

Data are presented as mean ± standard error of mean (SEM). The statistical significance of the difference between two groups of data was determined by Student’s t test. The significance of the differences among three groups of data were determined by ANOVA (analysis of variance) with post-hoc Tukey test.

## Supplementary Information

Supplementary Figures.

## Data Availability

The datasets used and/or analyzed during the current study are available from the corresponding authors on reasonable request.
