# Supplementary material for: σ2R/TMEM97 in retinal ganglion cell degeneration
Source: Sci Rep. 2022 Dec 1;12:20753. doi: 10.1038/s41598-022-24537-3 (PMC9715665; doi:10.1038/s41598-022-24537-3)
Supplement: Supplementary file 1 — Supplementary Figures. [file 41598_2022_24537_MOESM1_ESM.pdf]

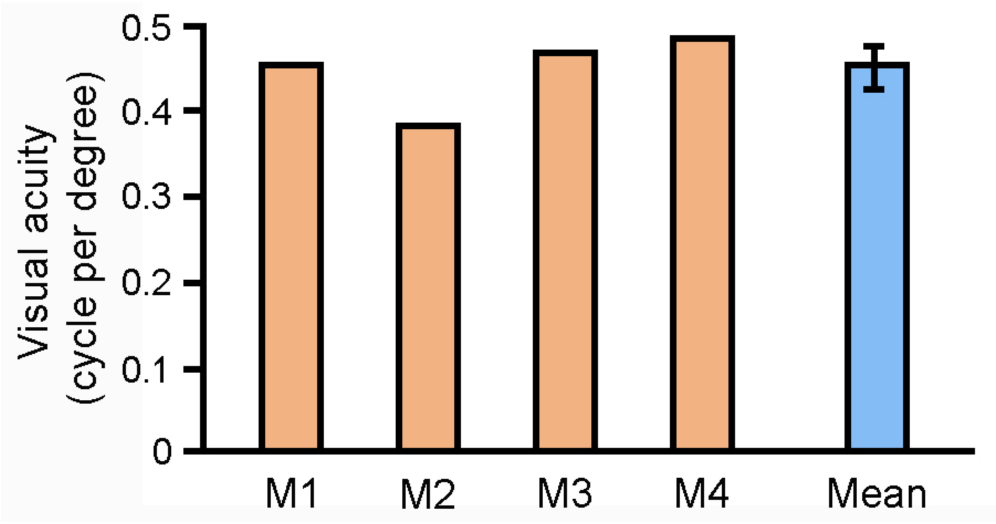

Supplementary Fig. S1. Visual acuity of *TMEM97*$^{-/-}$ mice. The visual acuity (VA) of each animal (M1-M4) was assessed with an optomotor system (yellow bars). The mean VA is 0.45±0.024 cpd (blue bar), in the range of wildtype mice.

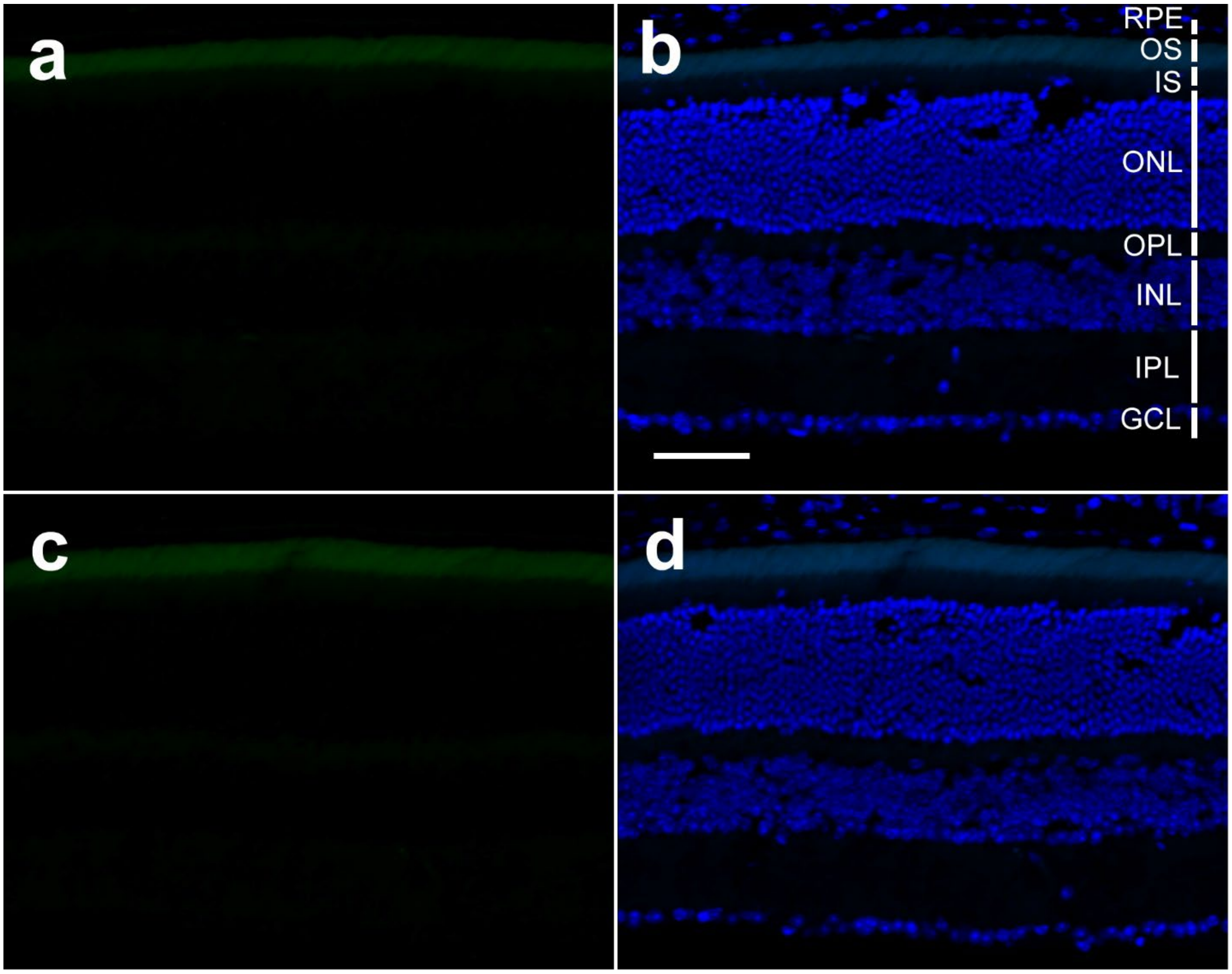

Supplementary Fig. S2. Absence of σ_2R/TMEM97 immunoreactivity in the retina of the *TMEM97*$^{-/-}$ mouse. Retinal sections from a *TMEM97*$^{-/-}$ mouse were stained for σ_2R/TMEM97 with the same antibodies used for immunostaining in wildtype mouse (Fig. 2**a)**. No specific σ_2R/TMEM97 immunoreactivity was detected (**a, b**). Weak autofluorescence was found in OS (**a, b**), which was also present in a retinal section without immunostaining (**c, d**). Retinal layers are indicated by white vertical bars in panel **b**. Scale bar: 100 µm.

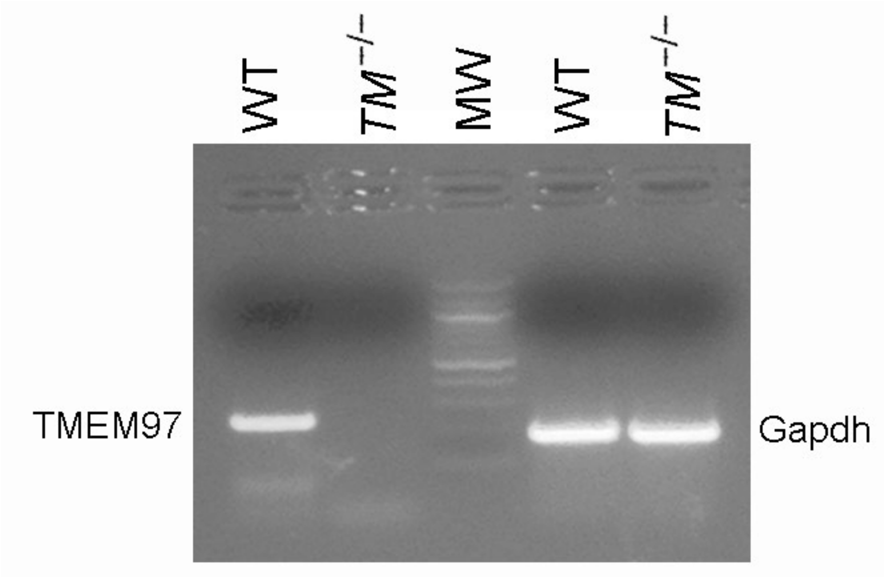

Supplementary Fig. S3. RNA expression of *TMEM97* in the retina. This is the full gel image of Fig. 2**d**. Robust *TMEM97* expression (TMEM97) was detected in the retinas from the wildtype animals (WT) but was absent in the retinas of *TMEM97*$^{-/-}$ mice (*TM*$^{-/-}$). Expression of Gapdh served as a reference. MW: molecular weight markers.

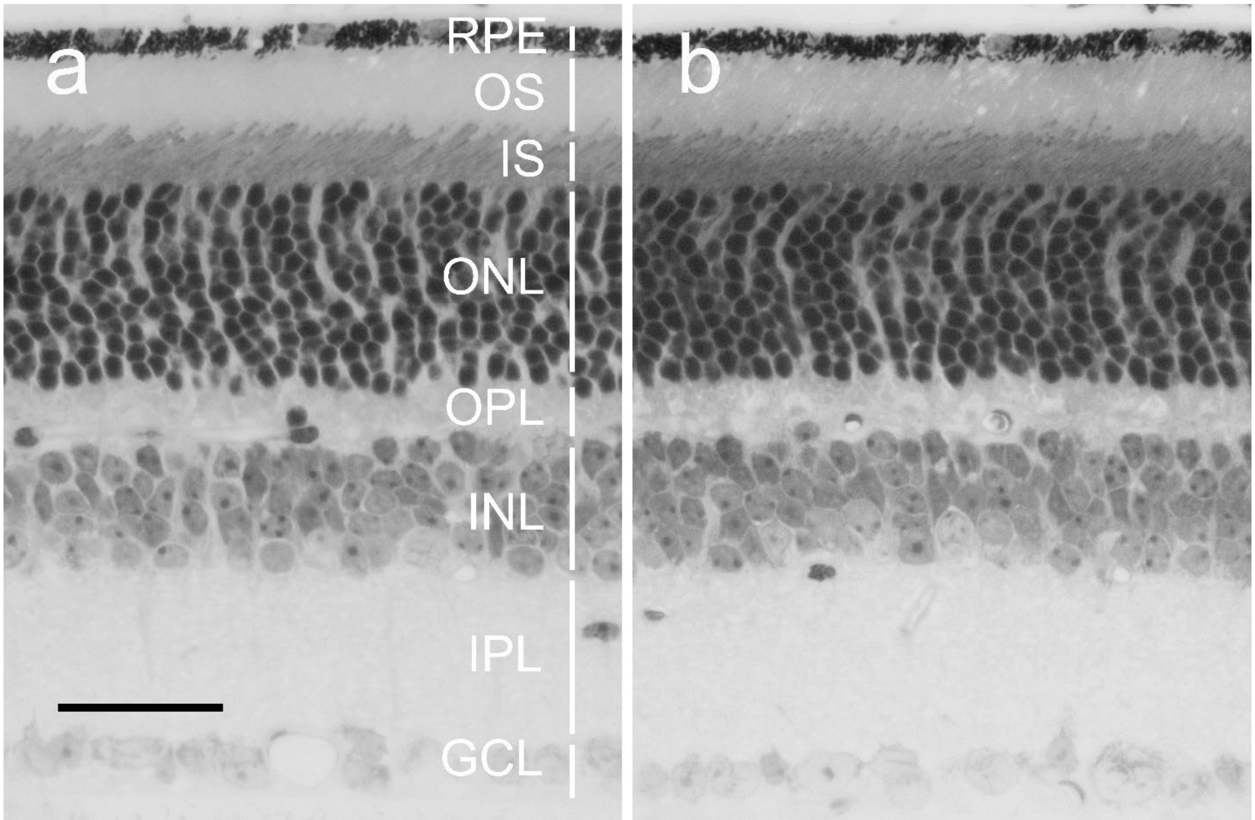

Supplementary Fig. S4. Retinal morphology after DKR-1677 treatment. No significant alteration of the overall retinal morphology is visible 5 days after intravitreal injection of DKR-1677 (2 μL DKR-1677 at 20 μg/μL) (**b**) as compared with the untreated fellow eye (**a**). Retinal layers are indicated by white vertical bars in panel **a**. Scale bar: 30 μm.
